# Comparative clinical evaluation of atlas and deep-learning-based auto-segmentation of organ structures in liver cancer

**DOI:** 10.1186/s13014-019-1392-z

**Published:** 2019-11-27

**Authors:** Sang Hee Ahn, Adam Unjin Yeo, Kwang Hyeon Kim, Chankyu Kim, Youngmoon Goh, Shinhaeng Cho, Se Byeong Lee, Young Kyung Lim, Haksoo Kim, Dongho Shin, Taeyoon Kim, Tae Hyun Kim, Sang Hee Youn, Eun Sang Oh, Jong Hwi Jeong

**Affiliations:** 1 Department of Radiation Oncology, Proton Therapy Center, National Cancer Center, 323, Ilsan-ro, Ilsandong-gu, Goyang-si, Gyeonggi-do 10408 South Korea; 2 Peter MacCallum Cancer Centre, Melbourne, VIC Australia; 3 Department of Radiation Oncology, Asan Medical Center, Seoul, South Korea; 4 Department of Radiation Oncology, Chonnam National University Medical School, Gwangju, South Korea

**Keywords:** Contouring, Atlas-based auto-segmentation, Deep-learning-based auto-segmentation, Deep convolution neural network (DCNN)

## Abstract

**Background:**

Accurate and standardized descriptions of organs at risk (OARs) are essential in radiation therapy for treatment planning and evaluation. Traditionally, physicians have contoured patient images manually, which, is time-consuming and subject to inter-observer variability.

This study aims to a) investigate whether customized, deep-learning-based auto-segmentation could overcome the limitations of manual contouring and b) compare its performance against a typical, atlas-based auto-segmentation method organ structures in liver cancer.

**Methods:**

On**-**contrast computer tomography image sets of 70 liver cancer patients were used, and four OARs (heart, liver, kidney, and stomach) were manually delineated by three experienced physicians as reference structures. Atlas and deep learning auto-segmentations were respectively performed with MIM Maestro 6.5 (MIM Software Inc., Cleveland, OH) and, with a deep convolution neural network (DCNN). The Hausdorff distance (HD) and, dice similarity coefficient (DSC), volume overlap error (VOE), and relative volume difference (RVD) were used to quantitatively evaluate the four different methods in the case of the reference set of the four OAR structures.

**Results:**

The atlas-based method yielded the following average DSC and standard deviation values (SD) for the heart, liver, right kidney, left kidney, and stomach: 0.92 ± 0.04 (DSC ± SD), 0.93 ± 0.02, 0.86 ± 0.07, 0.85 ± 0.11, and 0.60 ± 0.13 respectively. The deep-learning-based method yielded corresponding values for the OARs of 0.94 ± 0.01, 0.93 ± 0.01, 0.88 ± 0.03, 0.86 ± 0.03, and 0.73 ± 0.09. The segmentation results show that the deep learning framework is superior to the atlas-based framwork except in the case of the liver. Specifically, in the case of the stomach, the DSC, VOE, and RVD showed a maximum difference of 21.67, 25.11, 28.80% respectively.

**Conclusions:**

In this study, we demonstrated that a deep learning framework could be used more effectively and efficiently compared to atlas-based auto-segmentation for most OARs in human liver cancer. Extended use of the deep-learning-based framework is anticipated for auto-segmentations of other body sites.

## Background

Accuracy and precision of the delineated target volumes and surrounding organs at risk (OARs) is critical in radiotherapy treatment processing. However, to-this-date, these segmentation-based delineations are completed manually by physicians in the majority of clinical cases, which is a time-consuming task associated with an increased workload. Consequently, the reproducibility of this process is not always guaranteed, and ultimately depends on the physician’s experience [1]. In addition, manual re-segmentation is often necessary owing to anatomical changes and/or tumor responses over the course of the radiotherapy.

As such, model-based [2, 3] and atlas-based [4–7] auto-segmentation methods have been developed to maximize the efficiency gain, and concurrently minimize inter-observer variation. Various model-based methods have been published. Specifically, Qazi et al. [3] demonstrated use of adaptive model-based auto-segmentation of the normal and target structures for the head and neck, and Chen et al. [2] showed that active shape model-based segmentation could yield accuracy improvements of the order of 10.7% over atlas-based segmentation for lymph node regions. In the last few years, machine learning technology has been actively applied to various medical fields, such as for cancer diagnosis [8–10], medical imaging [11], radiation treatment [11, 12], and pharmacokinetics [13]. The application of one of the deep learning models [14], the convolutional neural network (CNN) [15], has recently yielded remarkable results in medical image segmentation [16–20].

The main advantage of deep learning methods is that they automatically generate the most suitable model from given training datasets. Therefore, a comparative study of the accuracy of each model is required to use auto-segmentation in clinical practice. Recently, Lustberg et al. [21] compared the auto contouring results in five organ structures with the use of the prototype of a commercial deep-learning contouring program (Mirada DLC Expert, Mirada Medical Ltd., Oxford, United Kingdom) with those obtained from an atlas-based contouring program (Mirada Medical Ltd., Oxford, United Kingdom).

In this study, we used the open source deep learning library, Keras (where the model can be loaded into the Tensorflow backend) instead of the commercial program. In addition, our neural network is based on Fusion net, an extension of the U-net suitable for medical image segmentation. This study aims to evaluate the clinical feasibility of an open source deep learning framework, using 70 liver cancer patients by comparing its performance against a commercially available atlas-based auto-segmentation framework.

## Methods

### Clinical datasets

Seventy patients with liver cancer diagnosed at the National Cancer Center in South Korea between the year of 2016–2017 were included in this study. All patients were treated with proton therapy, using 10 fractions of 660 or 700 cGy, with respective total doses of 6600 cGy and 7000 cGy. The characteristics of the patients are listed in Table 1. All computer tomography (CT) images were acquired using a General Electric (GE) Light speed radiotherapy (RT) system (GE Medical Systems, Milwaukee, WI). We used abdominal CT images with, the following dimensions for each axial slice: image matrix = 512 × 512, slice numbers = 80–128, pixel spacing = 1.00–1.04 mm, and slice thickness = 2.50 mm. Manually segmented contours for each organ were delineated by three senior expert physicians, and included segmentations of the heart, liver, kidney (left, right), and stomach. Manually segmented contours included the organ contours of the heart, liver, kidney (left, right), and stomach, which were mutually accepted by the three senior physicians following a joint discussion.

**Table 1:** Patient characteristics in this study

Patients	Male	Female	Average age	Location of liver cancer lesion								
				S1	S2	S3	S4	S5	S6	S7	S8	M
Training set	52	8	67	2	1	3	6	6	2	5	24	11
Testing set	8	2	69	–	–	1	1	1	1	–	3	3

*M:* Multiple lesion location

The study protocol conformed to the ethical guidelines of the Declaration of Helsinki as revised in 1983, and was approved by institutional review board (IRB) of National Cancer Center without IRB number. All patient data has been fully anonymized, and all methods were performed in accordance with the relevant guidelines and regulations outlined by our institution.

### Deep convolutional neural network

**Fig. 1:**
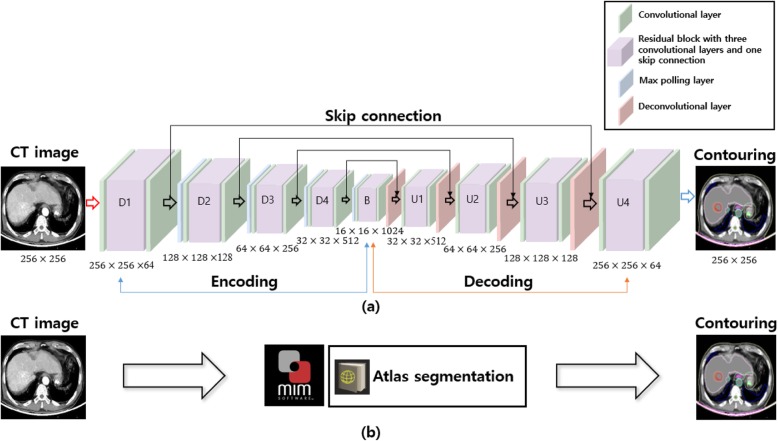
Segmentation of two-dimensional computer tomography (2D-CT) slice image using a (**a**) Fusion-Net-based deep convolutional neural network, **b** Atlas segmentation of MIM software. (conv: convolutional layer, res: residual layer, drop: dropout layer, batchnorm: batch normalization, max: maximum pooling layer, deconv: deconvolutional layer, merge: addition with the feature map from the encoding path by using a skip connection)

The network used was based on the open-source library Keras (version 2.2.4) [22] and the reference implementation of Fusion Net [23]. This network is a deep neural network which was developed based on the application of a residual CNN as an extension of U-net [24] to enable more accurate end-to-end image segmentation. It consists of a down-sampling (encoding) path and an up-sampling (decoding) path, as shown in Fig. 1. On the encoding path, we used a residual block layer (three convolution layer and one skip connection) between the two 3 × 3 convolution layers. Each of these layers was followed by a rectified linear unit (ReLu) [25], and one maximum pooling. On the decoding path, we used a 2 × 2 transposed convolution and a residual block layer between the two 3 × 3 convolution layers followed by a ReLu activation function. To avoid overfitting during the training stage, batch normalization [26] and dropout [27] were added to the layers. In the final layer, we used a 1 × 1 convolution network with a sigmoid activation function and a dice similarity coefficient loss function [28]. We used Adam [29] as an optimizer with the following training parameters: a learning rate of 1.0E-05, mini-batch size of twelves images, and a weight decay. A more detailed specification of our deep neural network, such as the number of feature maps, their sizes and ingredients, are listed Table 2. The experiments were conducted on a computer workstation with an Intel i7 central processing unit (CPU) with a 24 GB main memory, and a computer unified device architecture (CUDA) library on the graphics processing unit (GPU) (NVIDIA GeForce TITAN-Xp with 12 GB of memory). Network training of the deep convolutional neural network (DCNN) took approximately 48 h to run 2000 epochs on the training and validation datasets.

**Table 2:** Architecture of the proposed convolutional neural network

Block type	Ingredients	Size of feature maps
Input	–	256 × 256 ×1
Down layer (D1)	conv+res + drop+conv+batchnorm+max	128 ×128 × 64
Down layer (D2)	conv+res + drop+conv+batchnorm+max	64 ×64 × 128
Down layer (D3)	conv+res + drop+conv+batchnorm+max	32 ×32× 256
Down layer (D4)	conv+res + drop+conv+batchnorm+max	16 ×16 × 512
Bridge layer (B)	conv+res + conv	16 ×16 × 1024
Upscaling layer (U1)	deconv+merge+conv+res + conv	32 × 32 × 512
Upscaling layer (U2)	deconv+merge+conv+res + conv	64 × 64 × 256
Upscaling layer (U3)	deconv+merge+conv+res + conv	128 × 128 × 128
Upscaling layer (U4)	deconv+merge+conv+res + conv	256 × 256 × 64
Output	conv	256 × 256 ×1

### Segmentation image preprocessing

**Fig. 2:**
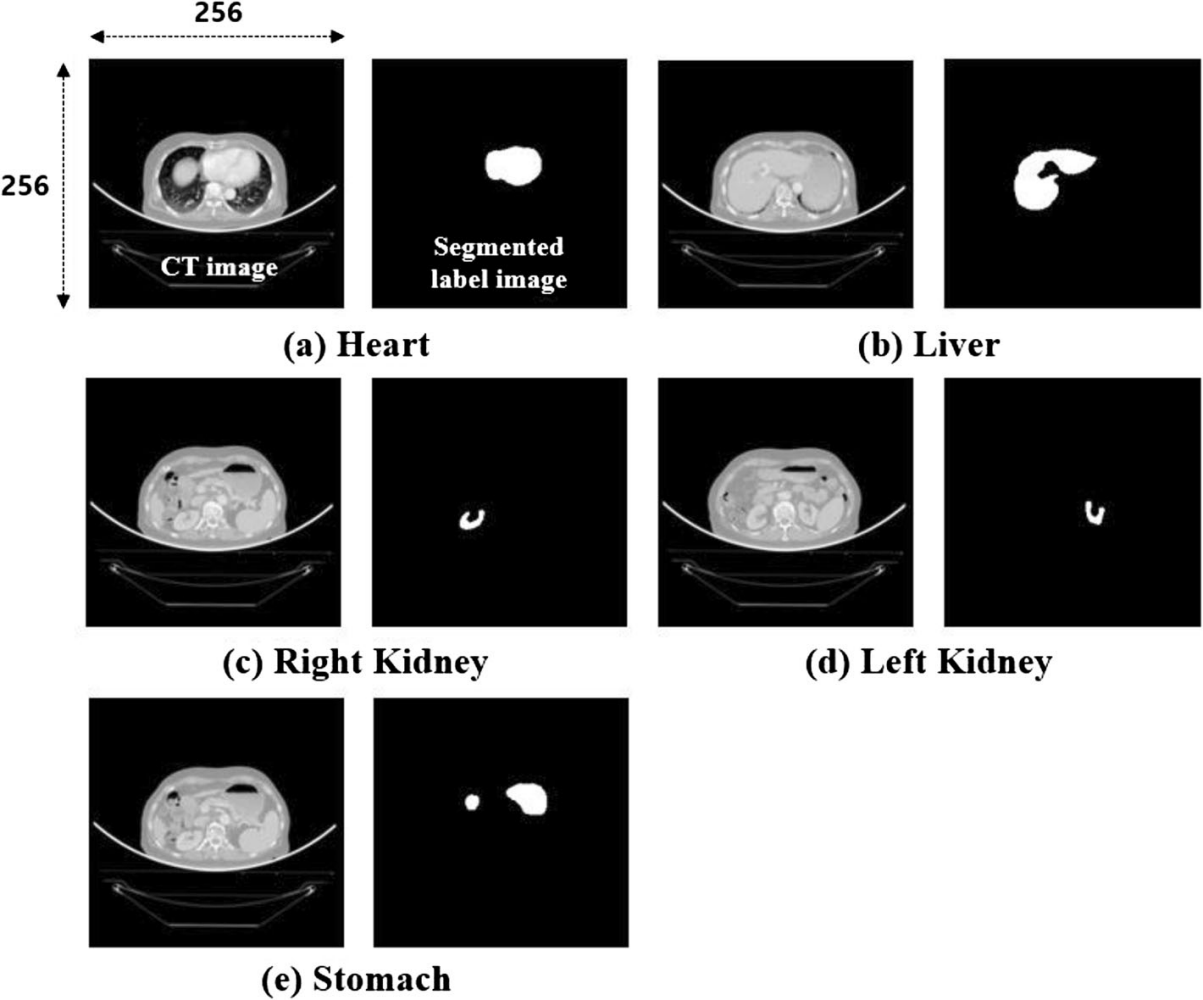
Grayscale CT and segmented label images of the (**a**) heart (H), **b** liver (L), **c** right kidney (RK), **d** left kidney (LK), and **e** stomach (S) used for DCNN model learning

CT planning images from patients and the required contouring information used for training of the DCNN were obtained using the Eclipse planning software (version 13.6, Varian Oncology Systems, Palo Alto, CA, USA). All CT images were converted to grayscale images, and the contouring points were converted to segmented label images in a binary format, as shown in Fig. 2. Hounsfield unit (HU) values were windowed in the range of − 100–600 to exclude irrelevant organs All images were downsampled from the conventional size of 512 × 512 pixels to the size of 256 × 256 pixels owing to graph card memory resource limitations and reduced DCNN training time constraints.

### Deep-learning-based segmentation

The deep-learning-based segmentation process consisted of three steps. The first was the random separation into training and validation sets consisting of 45 and 15 patient datasets, respectively, and the preprocessing and preparation of 10 independent test dataset images for the deep convolutional neural network.

**Fig. 3:**
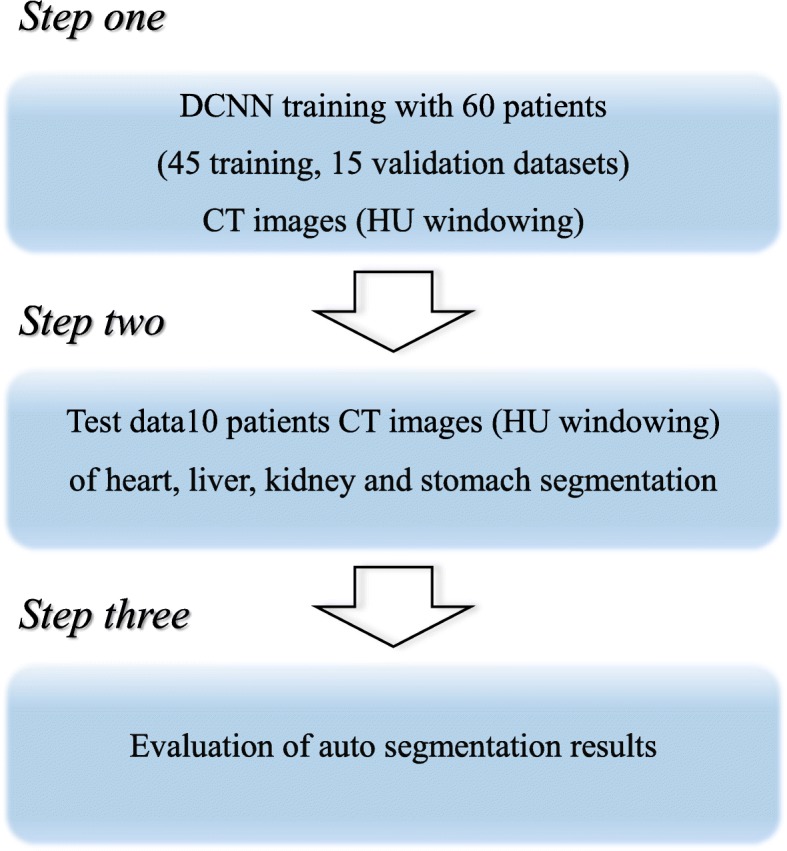
Work flowchart for deep convolution neural network (DCNN) training and testing

In the second step, we trained the DCNN using the training datasets for each of the organs. In the final step, the test image set was segmented into a test dataset with DCNN (Fig. 3).

### Atlas-based-segmentation

Atlas-based segmentation is a method used to locate the interface between the test image and the optimally matched organs from labeled, segmented reference image data [30].

The commercial atlas-based contouring software MIM Maestro 6.5 (MIM Software Inc., Cleveland, OH, USA) was used to generate the contours of the ten patients automatically test datasets for the OARs. Segmentation processing was performed on a single organ basis instead of multiple organ segmentation, and the outcomes were compared with those of the deep-learning-based segmentation conducted using the same conditions. We used MIM supported label fusion algorithms based on the majority vote (MV) algorithm.

For segmentation, a training set with data from 60 patients was registered to the MIM Maestro 6.5 atlas library with CT planning images alongside the respective manual contours of the heart, liver, kidney, and stomach. The slice thicknesses of the CT images were not changed during their registration with the atlas library.

### Quantitative evaluations of auto-segmentation

To quantitatively evaluate the accuracy of deep learning and atlas-based auto-segmentations, the Dice similarity coefficient (DSC) and Hausdorff distance (HD) were used for quantitative analyses on accuracy [31]. The DSC method, calculates the overlapping results of two different volumes according to the equation,

1 $$ DSC\ \left( dice\ similarity\ coefficient\right)=\frac{2\left|A\cap B\right|}{\left|A\right|+\left|B\right|}, $$

where A is the manual contouring volume, and B is the auto-segmentation volume (deep learning and atlas segmentation results). DSC takes values between zero and one. When the DSC value approaches zero, the manual and auto-segmentation outcomes differ significantly. However, as the DSC value approaches unity, the two-volumes exhibit increased similarities.

The second method is the HD method. After calculating the Euclidean distance of the surfaces of each contour point between A and B, the similarity of A and B is determined according to the distance of the nearest maximum distance. HD is thus defined as,

2 $$ Hausdorff\ distance\ (HD)=\mathit{\max}\ \left(h\left(A,B\right),h\left(B,A\right)\right), $$

where h(A, B) is the directed HD from A to B and is given by.

3 $$ h=\mathit{\max}\left(\min \left(\left\Vert a-b\right\Vert \right)\right) $$

$$ \mathrm{a}\in \mathrm{A},\mathrm{b}\in \mathrm{B} $$

As the HD approaches zeros, the difference between the manual contouring and auto contouring becomes smaller. By contrast, if the coefficient is greater than zero, the similarity between the two volumes decreases.

The third method is the volume overlap error (VOE) [32]. VOE can be calculated by subtracting the Jaccard coefficient from the value of unit by comparing dissimilarities between the two volumes.

$$ \mathrm{VOE}\ \left(\mathrm{volume}\ \mathrm{overlap}\ \mathrm{error}\right)=1-\frac{\left|A\cap B\right|}{\left|A\cup B\right|}, $$

The last method is the relative volume difference (RVD) [32]. RVD compares the sizes between two volumes.

$$ \mathrm{RVD}\ \left(\mathrm{relative}\ \mathrm{volume}\ \mathrm{difference}\right)=\frac{\left|B\right|-\left|A\right|}{\left|A\right|}, $$

Contrary to DSC, as the VOE and RVD approach zero, the manual contouring and auto contouring volumes only yield small volume differences, and values larger than zero reduce the similarity between the two volumes.

## Results

**Fig. 4:**
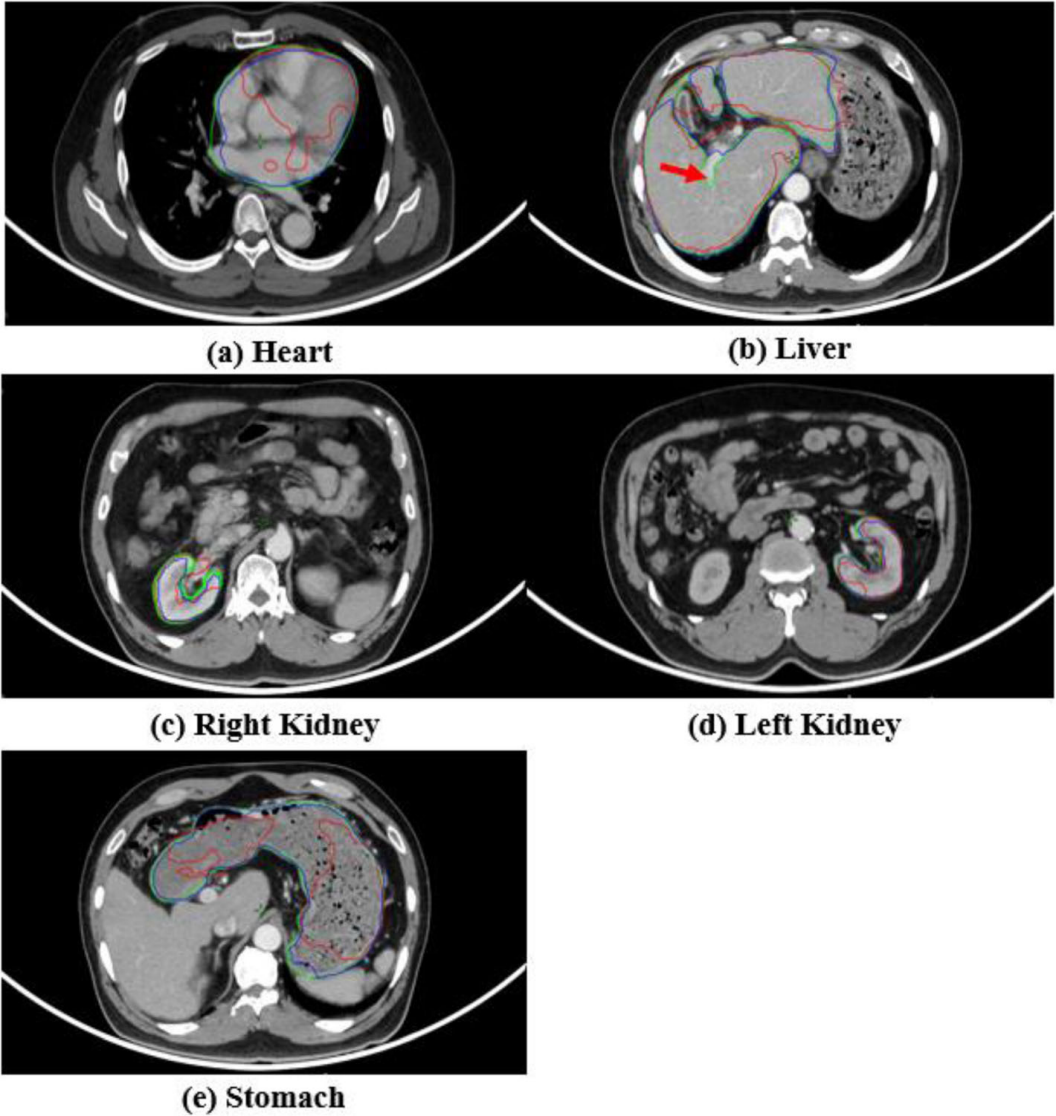
Selected CT slices of one of the studied patients with a manual contour (green), atlas-based contour (red), and deep-learning-based contour (blue) for the (**a**) heart, (**b**) liver, (**c**) right kidney, (**d**) left kidney, and (**e**) stomach

For quantitative evaluations, the DSC and HD were calculated for each test dataset, and the results are shown in Tables 3 and 4. For qualitative visual assessment, Fig. 4 shows, a specific patient case where the three delineation methods are compared, i.e., the atlas-based (C_atlas_), deep-learning-based (C_deep_), and manual contouring methods (C_manual_). In all the organ cases studied herein (i.e., heart, liver, kidney, and stomach), the C_deep_ results more accurate matched to the C_manual_ compared to the C_atlas_ results. However, both C_atlas_ and C_deep_ were not excluded in the hepatic artery region (Fig. 4, red arrow). For the kidney case, neither the C_atlas_ nor the C_deep_ outcomes differed significantly from the evoked C_manual_ outcomes from DSC.

**Table 3:** Comparison of dice similarity coefficients (DSC) obtained from atlas and deep-learning-based segmentations in the cases of the four tested organs (heart, liver, kidney, stomach). Averages and standard deviations are listed for all the ten tested cases

Test Case	Heart		Liver		Right kidney		Left kidney		Stomach	
	C_atlas_	C_deep_	C_atlas_	C_deep_	C_atlas_	C_deep_	C_atlas_	C_deep_	C_atlas_	C_deep_
# 1	0.95	0.96	0.93	0.93	0.85	0.86	0.78	0.83	0.41	0.80
# 2	0.93	0.93	0.92	0.93	0.93	0.92	0.93	0.88	0.78	0.71
# 3	0.96	0.95	0.95	0.94	0.87	0.88	0.61	0.84	0.58	0.71
# 4	0.96	0.96	0.95	0.94	0.85	0.86	0.93	0.89	0.53	0.57
# 5	0.92	0.93	0.94	0.93	0.84	0.89	0.89	0.78	0.63	0.88
# 6	0.85	0.94	0.89	0.92	0.89	0.89	0.91	0.88	0.61	0.79
# 7	0.85	0.94	0.90	0.93	0.95	0.93	0.94	0.86	0.79	0.72
# 8	0.86	0.93	0.92	0.93	0.91	0.84	0.92	0.85	0.74	0.83
# 9	0.96	0.94	0.92	0.93	0.70	0.84	0.88	0.88	0.38	0.61
# 10	0.91	0.93	0.94	0.94	0.78	0.84	0.70	0.88	0.56	0.64
Avg SD	0.92	0.94	0.93	0.93	0.86	0.88	0.85	0.86	0.60	0.73
	0.04	0.01	0.02	0.01	0.07	0.03	0.11	0.03	0.13	0.09

*Avg: * Average

*SD: * Standard deviation

**Table 4:** Comparison of Hausdorff distances (HD) for atlas against deep-learning-based segmentation for the with four organs (heart, liver, kidney, stomach). Averages and standard deviations are listed for ten tested cases

Test Case	Heart		Liver		Right kidney		Left kidney		Stomach	
	C_atlas_	C_deep_	C_atlas_	C_deep_	C_atlas_	C_deep_	C_atlas_	C_deep_	C_atlas_	C_deep_
# 1	1.06	1.15	1.90	1.89	1.03	1.80	2.26	1.49	8.88	3.47
# 2	1.36	1.88	1.79	1.72	1.14	1.29	0.95	1.51	3.09	3.57
# 3	0.66	1.45	1.37	2.85	1.35	1.69	4.82	1.79	6.70	5.54
# 4	0.65	1.23	2.05	2.16	0.55	0.52	0.85	1.58	7.69	5.35
# 5	1.53	1.46	1.34	1.56	1.16	1.95	1.22	2.37	6.83	2.65
# 6	4.70	1.80	4.09	2.27	1.59	1.95	1.33	2.04	8.35	5.99
# 7	4.70	1.63	2.84	1.84	0.76	1.20	1.02	2.37	3.35	4.62
# 8	3.47	1.59	2.06	2.31	1.46	1.32	1.00	1.77	4.58	3.38
# 9	0.92	1.80	3.05	2.63	4.66	2.48	2.10	1.80	10.67	5.89
# 10	2.51	2.10	1.83	2.45	4.11	1.91	3.45	2.10	7.42	8.14
Avg SD	2.16	1.61	2.23	2.17	1.78	1.61	1.90	1.88	6.76	4.86
	1.52	0.28	0.81	0.39	1.34	0.52	1.24	0.31	2.31	1.57

*Avg: * Average

*SD: * Standard deviation

The methods of auto-segmentation were quantitatively compared using DSC, HD, VOE and RVD metrics against manual contours (i.e., the reference), and are presented in Tables 3, 4, 5, and 6, respectively.

**Table 5:** Comparison of volume overlap error (VOE) for atlas-based segmentation against deep-learning-based segmentation with four organs (heart, liver, kidney, stomach). Averages and standard deviations are listed for ten test cases

Test Case	Heart		Liver		Right kidney		Left kidney		Stomach	
	C_atlas_	C_deep_	C_atlas_	C_deep_	C_atlas_	C_deep_	C_atlas_	C_deep_	C_atlas_	C_deep_
# 1	8.49	6.83	10.22	10.58	12.88	10.31	30.15	18.37	84.83	31.01
# 2	11.68	13.24	9.95	7.81	17.62	18.38	14.39	19.62	45.96	39.11
# 3	7.34	11.17	8.39	13.44	12.42	11.07	63.90	12.42	65.73	34.86
# 4	11.17	12.73	6.73	7.34	8.86	8.54	8.34	10.31	81.17	54.12
# 5	13.87	11.89	10.29	11.64	9.39	7.59	20.76	35.07	72.42	31.16
# 6	26.60	11.95	34.32	15.32	15.17	15.66	14.32	17.84	55.58	35.68
# 7	24.39	6.66	20.41	11.02	8.10	9.03	9.99	12.00	32.21	41.44
# 8	19.34	12.12	13.29	10.30	15.27	15.41	16.58	12.53	39.02	23.24
# 9	7.74	10.34	13.14	12.07	40.66	13.06	20.15	14.59	85.51	42.75
# 10	21.04	11.48	8.37	8.68	34.68	12.87	57.73	10.39	63.92	41.97
Avg SD	15.17	10.84	13.51	10.82	17.51	12.19	25.63	16.31	62.64	37.53
	6.77	2.18	7.83	2.36	10.57	3.33	18.57	7.01	18.10	7.99

*Avg:* Average

*SD:* Standard deviation

**Table 6:** Comparison of relative volume difference (RVD) for atlas-based segmentation against deep-learning-based segmentation with four organs (heart, liver, kidney, stomach). Averages and standard deviations are listed for ten test cases

Test Case	Heart		Liver		Right kidney		Left kidney		Stomach	
	C_atlas_	C_deep_	C_atlas_	C_deep_	C_atlas_	C_deep_	C_atlas_	C_deep_	C_atlas_	C_deep_
# 1	0.78	1.10	3.22	2.44	4.63	0.36	14.9	2.67	84.38	14.56
# 2	7.65	9.33	12.10	0.89	3.57	1.77	6.27	0.12	21.33	18.52
# 3	3.08	8.51	0.70	3.81	9.17	3.39	20.25	1.44	48.75	25.55
# 4	7.40	5.13	3.30	0.89	1.62	0.51	0.60	1.26	59.02	19.12
# 5	15.48	0.62	2.89	1.16	1.18	1.58	1.56	7.57	63.49	10.94
# 6	24.82	8.20	12.20	0.35	10.58	1.77	6.16	1.55	70.37	43.71
# 7	23.57	1.77	13.21	0.81	2.11	2.35	3.94	2.69	25.68	30.79
# 8	11.80	7.51	0.11	0.83	4.52	9.72	12.15	3.51	21.67	14.32
# 9	8.31	2.74	4.66	3.64	42.26	12.9	12.29	2.49	89.19	20.80
# 10	26.12	6.75	3.18	3.81	17.87	10.93	24.21	1.21	16.73	14.30
Avg SD	12.90	5.17	5.56	1.86	9.75	4.53	10.23	2.45	50.06	21.26
	8.72	3.17	4.72	1.34	11.89	4.49	7.52	1.94	25.92	9.35

*Avg:* Average

*SD:* Standard deviation

The average DSC values (± SD) of C_atlas_ are 0.92 (±0.04), 0.93 (±0.02), 0.86 (±0.07), 0.85 (±0.11), and 0.60 (±0.13) for the heart, liver, right kidney, left kidney, and stomach, respectively. The respective outcomes for the same DSC analyses for C_deep_ are 0.94 (±0.01), 0.93 (±0.01), 0.88 (±0.03), 0.86 (±0.03), and 0.73 (±0.09), for the heart, liver, right kidney, left kidney, and stomach, respectively.

**Fig. 5:**
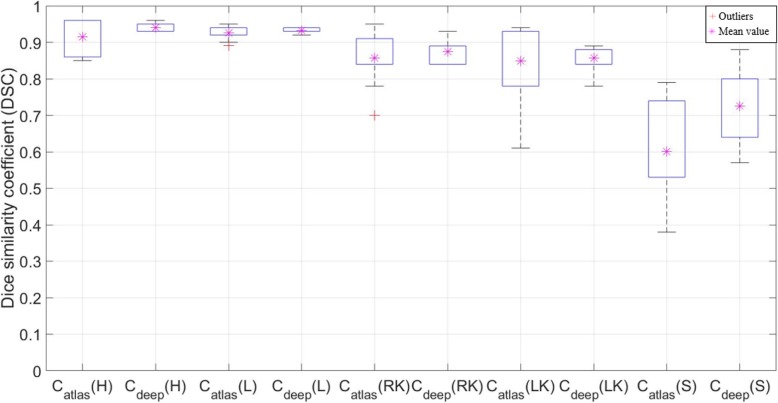
Comparison of Hausdorff distances (HD) for deep learning contour (C_deep_) and atlas-based contour (C_atlas_) segmentations for the heart (H), liver (L), right kidney (RK), left kidney (LK), and stomach (S)

**Fig. 6:**
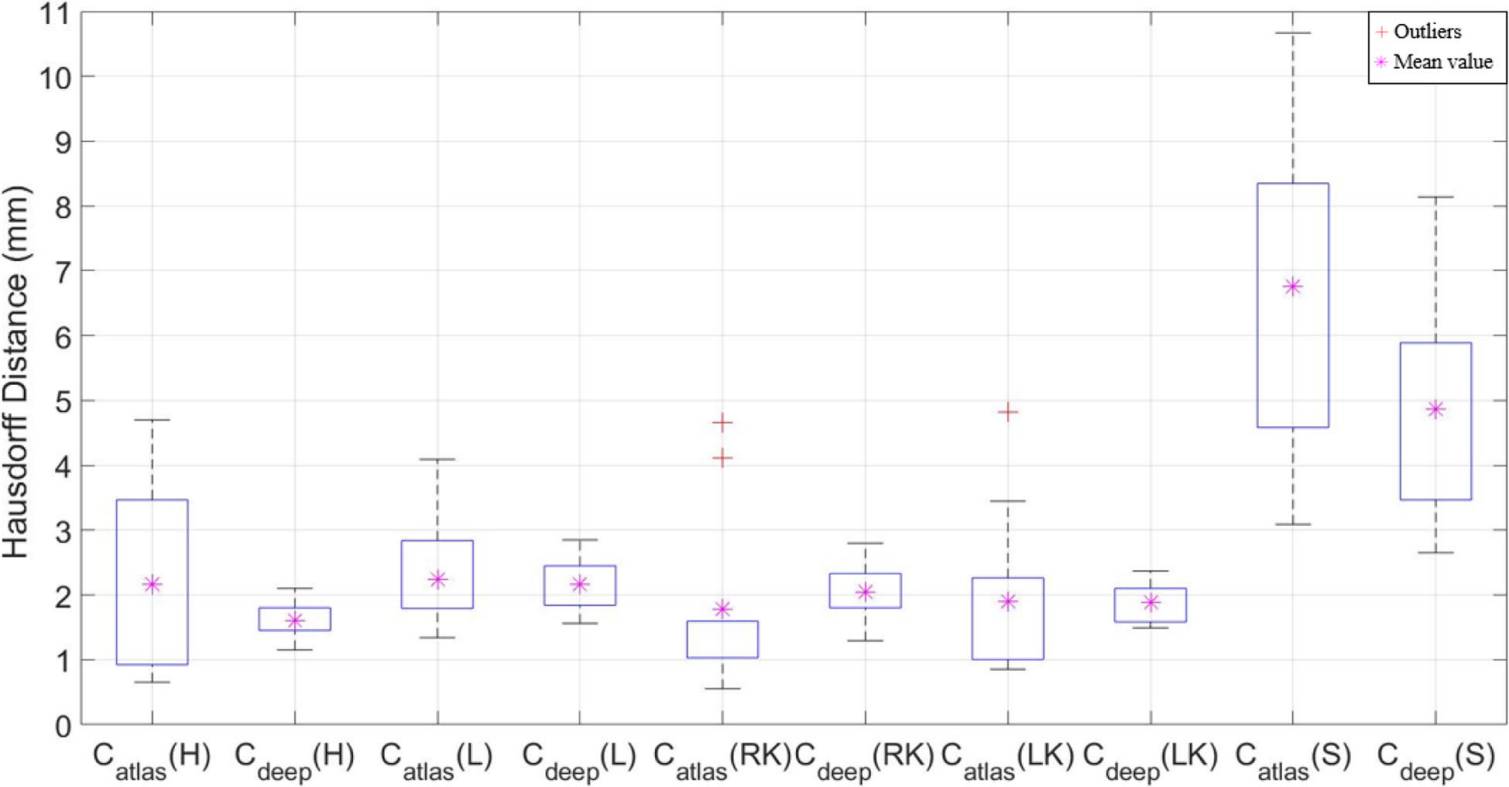
Comparison of Dice similarity coefficient (DSC) value of deep learning contour (C_deep_) and atlas-based contour (C_atlas_) segmentations for the heart (H), liver (L), right kidney (RK), left kidney (LK), and stomach (S)

The HD values (± SD) for C_atlas_ are 2.16 (±1.52) mm, 2.23 (±0.81) mm, 1.78 (±1.34) mm, 1.90 (±1.24) mm, and 6.76 (±2.31) mm, for the heart, liver, right kidney, left kidney, and stomach, respectively. The respective outcomes for the HD values based on the same analysis for C_deep_ are 1.61 (±0.28) mm, 2.17 (±0.39) mm, 1.61 (±0.52) mm, 1.88 (±0.31) mm, and 4.86 (±1.57) mm, for the heart, liver, right kidney, left kidney, and stomach, respectively, as shown in Fig. 6. The average DSC outcomes for C_deep_ are higher in all the cases except for the liver. Specifically, there was a maximum difference of 21.67% in the stomach case, as shown in Table 8. It is important to note that the standard deviations of the DSC values for C_atlas_ were higher than those of C_deep_ for all the studied structures, i.e., C_atlas_ exhibits broader interquartile ranges than C_deep_ in the boxplot, as shown in Fig. 5.

**Table 7:** Differences between HD mean values associated with the deep learning and atlas-based contouring methods

Subject organs		Heart	Liver	Right Kidney	Left Kidney	Stomach
HD (mm)	C_deep_	1.61	2.17	1.61	1.88	4.86
	C_atlas_	2.16	2.23	1.78	1.90	6.76
C_deep_ – C_atlas_ (mm)		−0.55	−0.06	−0.17	−0.02	−1.90

**Fig. 7:**
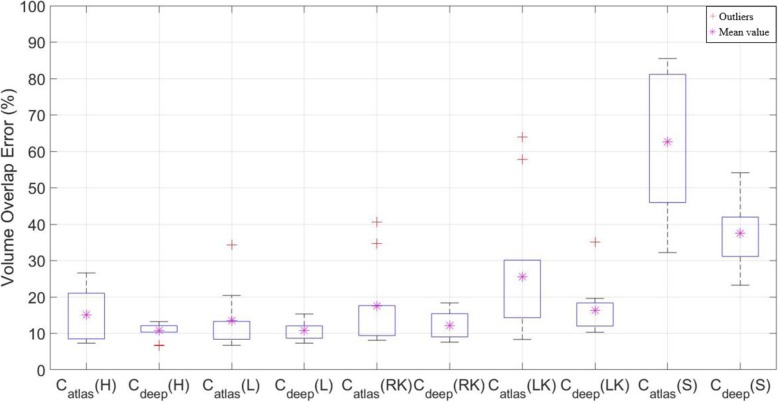
Comparison of volume overlap error (VOE) for deep learning contour (C_deep_) and atlas-based contour (C_atlas_) segmentations for the heart (H), liver (L), right kidney (RK), left kidney (LK), and stomach (S)

**Fig. 8:**
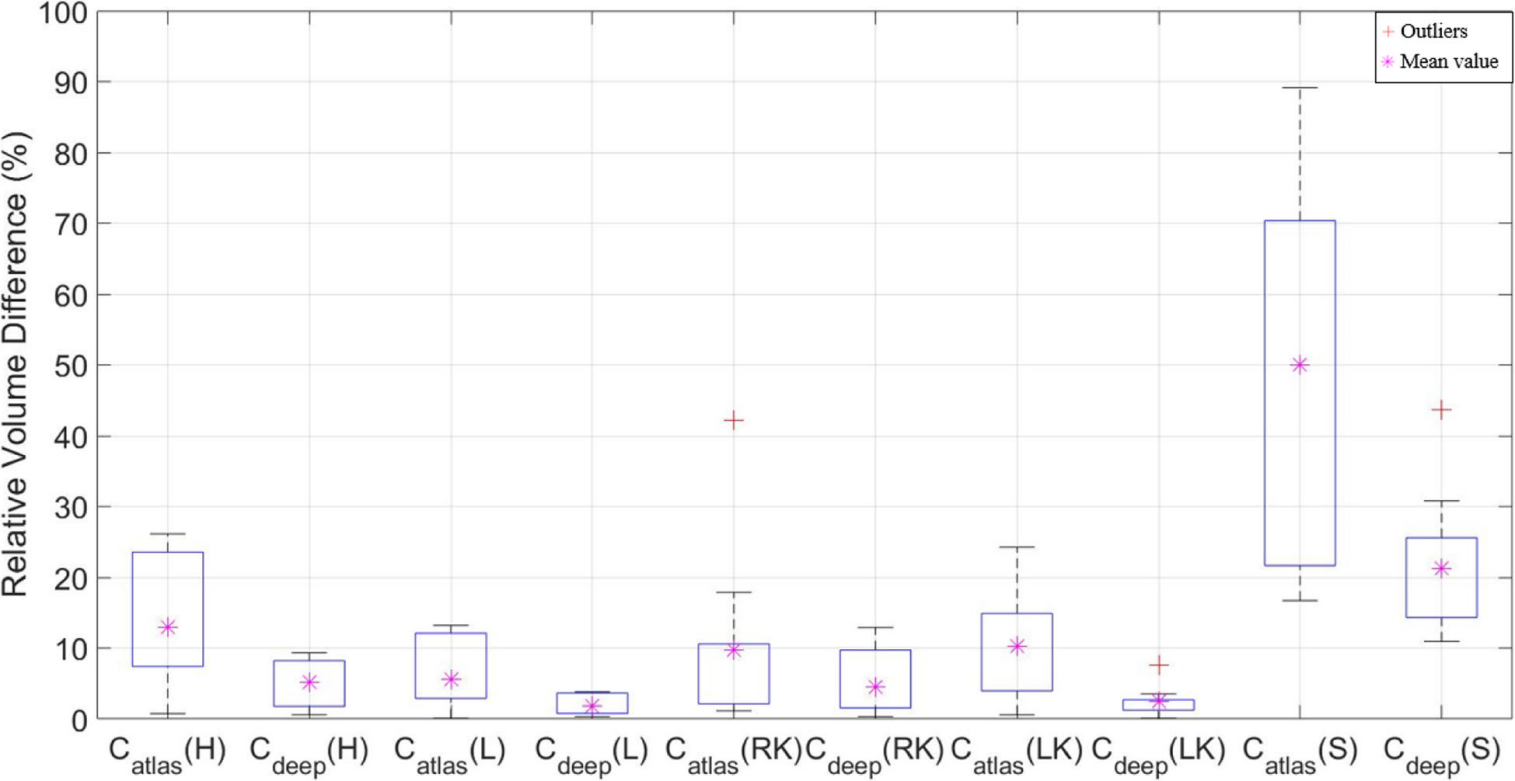
Comparison of relative volume difference (RVD) for deep learning contour (C_deep_) and atlas-based contour (C_atlas_) segmentations for the heart (H), liver (L), right kidney (RK), left kidney (LK), and stomach (S)

The VOE and RVD results showed significant differences between C_atlas_ and C_deep_ compared to DSC, as shown in Figs. 7 and 8. In Table 8, average of DSC results in the liver case were not different, but the VOE and RVD showed a more accurate difference of ~ 3%, and the heart, kidney (left, right) and stomach also showed significantly differences than DSC, as shown in Tables 9 and 10. In addition, Christ et al. [32] have also published a liver case auto segmentation study, whereby VOE and RVD yielded more sensitive differences compared to the DSC results.

**Table 8:** Differences between DSC mean values associated with the deep-learning and atlas-based contouring methods

Subject organs		Heart	Liver	Right Kidney	Left Kidney	Stomach
DSC	C_deep_	0.94	0.93	0.88	0.86	0.73
	C_atlas_	0.92	0.93	0.86	0.85	0.60
1- (C_deep_ / C_atlas_) (%)		−2.17	0	−2.33	−1.18	−21.67

**Table 9:** Differences between VOE mean values associated with the deep-learning-based and atlas-based contouring methods

Subject organs		Heart	Liver	Right Kidney	Left Kidney	Stomach
VOE (%)	C_deep_	10.84	10.82	12.19	16.31	37.53
	C_atlas_	15.17	13.51	17.51	25.63	62.64
C_deep_ – C_atlas_ (%)		−4.33	−2.69	−5.32	−9.32	−25.11

**Table 10:** Differences between RVD mean values associated with the deep-learning-based and atlas-based contouring methods

Subject organs		Heart	Liver	Right Kidney	Left Kidney	Stomach
RVD (%)	C_deep_	5.17	1.86	4.53	2.45	21.26
	C_atlas_	12.90	5.56	9.75	10.23	50.06
C_deep_ – C_atlas_ (%)		−7.73	−3.70	−5.22	−7.78	−28.80

## Discussion

In this study, 70 CT patient datasets (45 for training, 15 for validation, and 10 for testing) were used to compare the performances of the atlas-and deep-learning-based auto-segmentation frameworks. In the study of La Macchia et al. [33], the DSC results obtained from the auto-segmentation analyses for the heart, liver, left kidney and right kidney, with the use of the three commercially available systems (ABAS 2.0, MIM 5.1.1, and Velocity AI 2.6.2) were in the ranges of 0.87–0.88, 0.90–0.93, 0.81–0.89, and 0.83–0.89, respectively. The heart yielded lower DSC scores than our reported results, whereas the other organ cases were similar to our segmented results.

However, poorer performance outcomes were evoked in the case of the stomach compared to the other organs in terms of DSC owing to the fact that the performance of our method depended on the presence of gas bubbles and on the variation of the stomach shapes among the studied patient cases (Table 8). Nevertheless, as shown in Tables 7 and 8, it is important to note that the deep learning method yielded more accurate results both in terms of the DSC (by 21.67%) and HD (− 1.90 mm) compared to the atlas-based method.

The time-efficiency was based on the average times required by the atlas and deep-learning-based segmentation methods for the four organs, which were 75 s and 76 s, respectively (i.e., there was no statistically significant difference because *p*-values were larger than 0.05 when a ranked Wilcoxon test was performed).

However, in the case of the atlas-based segmentation, the time required for multi-organ segmentation can be reduced. A recent study by Gibson et al. [34] demonstrated a multi-organ segmentation approach using the deep learning framework. Our future studies will be undertaken based on the implementation of multi-organ segmentation using DCNN to investigate the impact of discrepancies among different segmentation methods in radiation treatment planning.

It is also important to note that this study is associated with some limitations. First, to compare the segmentation performances of the two methods using the same conditions, we did not use the image datasets which were obtained by cropping the relevant regions-of-interest [16]. Secondly, we did not perform post-image processing. Third, the number of test sets was only ten. Finally, the limitation associated with the use of our deep learning network, was based on the fact that the CT image was a three-dimensional (3D)-volume matrix, and each two-dimensional (2D) image was structurally connected to the previous image. However, DCNN does not take into account this structural connectivity because it uses a 2D convolution filter. All these factors may affect the performance of the auto-segmentation process. In post-image processing, Kim et al. [35] showed that the accuracy of the predicted contouring may vary differs according to the smoothing level of the contouring boundary surface. However, it would be difficult to represent statistically significant data for all clinical cases using such a small test dataset. In addition, recent studies have used 3D convolution filters to perform medical image segmentation. Milletari et al. [36] performed volumetric segmentation of magnetic resonance (MR) prostate images with a 3D volumetric CNN, an average dice score of 0.87 ± 0.03 and an average HD of 5.71 ± 1.02 mm.

The HD exhibited a difference in accuracy which depended on the image size. The size of the CT and segmented labeled images were reduced to half the original sizes (i.e., to 256 × 256) because of the limitations of the graphic card memory and training time constraints. The standard deviations (SD) of the HD results after image interpolation to the matrix sizes of 64 × 64 pixels, 128 × 128 pixels, and 512 × 512 pixels, compared to the current pixels array size 256 × 256 pixels were ± 0.63 mm, ± 0.58 mm, ± 0.97 mm, ± 0.90 mm, and ± 1.03 mm for the heart, liver, right kidney, left kidney, and stomach, respectively. Accordingly, when the segmentation image size is changed, the HD result may yield a difference up to approximately 1 mm.

However, comparison of the SD of the HD results of the current pixel array size (256 × 256) and the original CT pixel array size (512 × 512 pixels) yielded differences which were equal to ±0.02 mm, ± 0.04 mm, ± 0.04 mm, ± 0.07 mm, and ± 0.08, in the cases of the heart, liver, right kidney, left kidney, and stomach, respectively.

Despite the aforementioned limitations, in this study, we compared the auto segmentation outcomes obtained with the use of the atlas, which is the auto segmentation tool currently used in clinical practice, with the use of an open source-based tool [21] rather than the commercial program [20].

In particular, HD is a sensitive index which indicates whether segmentation yields localized disagreements. Therefore, it is an important indicator for assessing the accuracy of the segmented boundaries. Considering the limitation of the SD differences based on pixel array size differences (comparison of the array sizes of 256 × 256 and 512 × 512) mentioned above, the deep-learning-based contouring is superior to the atlas-based contouring method regarding the HD results.

The segmentation results of the heart, liver, kidney, and stomach, based on the use of the auto-segmentation with deep-learning-based contouring showed good performance outcomes both in terms of DSC and HD compared to the atlas-based contouring. Loi et al. [37] proposed a sufficient DSC threshold > 0.85 for volumes greater than 30 ml for auto-segmentations. In this study, the vast majority met this criterion except in the case of the stomach, whereby only one of the test sets yielded DSC values greater than 0.85 in the case where, the deep learning method was used (Table 3).

Recent technological developments in diagnostic imaging modalities have led to frequent fusions of images, including the paradigms of MR–Linac, PET–CT, and MR–CT image fusions. To apply this to adaptive RT, efficient OAR delineation is necessary in the daily adaptive treatment protocol to minimize the total treatment time.

There is one important issue that needs to be considered to contour the OARs correctly, which pertains to the motion artifacts attributed to the respiratory motion of the patients. The movement of the organ increases the contour uncertainty of the OARs. Combining the auto-segmentation with the reduction of motion artifacts [38] will enable more accurate delineation of the organs affected by respiration. Therefore, application of deep-learning-based auto-segmentation possesses tremendous potential, and is expected to have a greater impact in the near future in achieving effective and efficient radiotherapy workflow.

## Conclusions

In summary, we applied an open-source, deep learning framework to an auto-segmentation application in liver cancer and demonstrated its performance improvements compared to the atlas-based approach. Deep-learning-based auto-segmentation is considered to yield an acceptable accuracy as well as good reproducibility for clinical use. Additionally, it can significantly reduce the contouring time in OARs destined to undergo radiation treatment planning. We envisage that deep learning-based auto-segmentation will become clinically useful, especially when it is applied in the daily adaptive plans which are based on multi-imaging modality-guided treatments.

## Data Availability

The data are not available for public access because of patient privacy concerns, but are available from the corresponding author on reasonable request.

## Abbreviations

CNN: Convolutional neural network
CT: Computer tomography
DCNN: Deep convolution neural network
DSC: Dice similarity coefficient
HD: Hausdorff distance
MV: Majority vote
OARs: Organs at risk
ReLu: Rectified linear unit
RT: Radiotherapy
RVD: Relative volume difference
SD: Standard deviation
VOE: Volume overlap error
